# Spin-Orbit Coupling and Spin-Polarized Electronic Structures of Janus Vanadium-Dichalcogenide Monolayers: First-Principles Calculations

**DOI:** 10.3390/nano12030382

**Published:** 2022-01-24

**Authors:** Ming-Hao Lv, Chang-Ming Li, Wei-Feng Sun

**Affiliations:** 1 Key Laboratory of Engineering Dielectrics and Its Application, Ministry of Education, School of Electrical and Electronic Engineering, Harbin University of Science and Technology, Harbin 150080, China; lemenhuene@gmail.com (M.-H.L.); kingstel@163.com (C.-M.L.); 2 School of Electrical and Electronic Engineering, Nanyang Technological University, Singapore 639798, Singapore

**Keywords:** phonon structure, electronic structure, spin polarization, spin-orbit coupling, first-principles calculation

## Abstract

Phonon and spintronic structures of monolayered Janus vanadium-dichalcogenide compounds are calculated by the first-principles schemes of pseudopotential plane-wave based on spin-density functional theory, to study dynamic structural stability and electronic spin-splitting due to spin-orbit coupling (SOC) and spin polarization. Geometry optimizations and phonon-dispersion spectra demonstrate that vanadium-dichalcogenide monolayers possess a high enough cohesive energy, while VSTe and VTe_2_ monolayers specially possess a relatively higher in-plane elastic coefficient and represent a dynamically stable structure without any virtual frequency of atomic vibration modes. Atomic population charges and electron density differences demonstrate that V–Te covalent bonds cause a high electrostatic potential gradient perpendicular to layer-plane internal VSTe and VSeTe monolayers. The spin polarization of vanadium 3*d*-orbital component causes a pronounced energetic spin-splitting of electronic-states near the Fermi level, leading to a semimetal band-structure and increasing optoelectronic band-gap. Rashba spin-splitting around G point in Brillouin zone can be specifically introduced into Janus VSeTe monolayer by strong chalcogen SOC together with a high intrinsic electric field (potential gradient) perpendicular to layer-plane. The vertical splitting of band-edge at K point can be enhanced by a stronger SOC of the chalcogen elements with larger atom numbers for constituting Janus V-dichalcogenide monolayers. The collinear spin-polarization causes the band-edge spin-splitting across Fermi level and leads to a ferrimagnetic order in layer-plane between V and chalcogen cations with higher *α* and *β* spin densities, respectively, which accounts for a large net spin as manifested more apparently in VSeTe monolayer. In a conclusion for Janus vanadium-dichalcogenide monolayers, the significant Rashba splitting with an enhanced K-point vertical splitting can be effectively introduced by a strong SOC in VSeTe monolayer, which simultaneously represents the largest net spin of 1.64 (*ћ*/2) per unit cell. The present study provides a normative scheme for first-principles electronic structure calculations of spintronic low-dimensional materials, and suggests a prospective extension of two-dimensional compound materials applied to spintronics.

## 1. Introduction

Spin-orbit coupling (SOC) plays a crucial role in the intensively studied quantum systems, such as topological insulators, Swinger, and Rashba materials. When SOC resides substantially in the atom periodic system without inversion symmetry, the effective magnetic fields arise to cause various physical effects, such as current-induced spin polarization, spin-Hall effect, spin-electric coupling and spin-ballistic transport, which account for the emergence of diverse practical spintronic devices [1,2,3,4]. In particular, the spintronic applications of strong SOC low-dimensional materials make an immense progress in the implement of room-temperature-operating spintronic devices [5,6].

Characterized by two-dimensional systems with strong SOC, the transition-metal dichalcogenides (TMD) have gathered attention for their potential flexible exploitation in low-power electronic, spintronic and optoelectronic devices. Because of their intrinsic band-gap of about 1.1–1.9 eV, TMD monolayers are considered good candidates for the channel materials in field-effect transistors (FETs), as well as promising materials for optoelectronics [7,8,9,10]. In addition, the broken inversion symmetry together with the giant spin-orbit coupling (SOC) that originates from the *d* orbitals of metal atoms in TMD monolayers, induces a large spin splitting approaching to ~500 meV at high symmetry points of hexagonal Brillouin zone [11,12]. The strong coupling between spin and valley degrees of freedom makes TMD monolayers ideal valleytronic materials [13,14]. In contrast to non-polar MX_2_ monolayers, the polar two-dimensional systems of Janus MXY (M = Mo, W; X ≠ Y = S, Se, Te) monolayers represent a considerable Rashba SOC-induced spin splitting at Brillouin zone center for the highest valence-band, which derives from the asymmetric electrostatic potential (out-of-plane intrinsic electric field) caused by breaking mirror symmetry [15]. Rashba SOC can be effectively ameliorated by applying an in-plane biaxial strain to modify chalcogen bonding and intrinsic electric field [16]. In contrast to III–VI double layers or MXY heterostructures with a Rashba splitting deriving from the surface or interface electronic states under applying electric fields [17,18], the Janus TMD monolayers lacking out-of-plane mirror symmetry may present Rashba splitting from internal electric field and strong SOC of heavy chalcogens, which facilitate their application in spintronic devices. Furthermore, Janus TMD monolayers exhibit the transcendental characteristics of large out-of-plane piezoelectric performance, high hydrogen hydrocatalytic capability and broad absorption in solar spectrum, all of which can be attributed to to the imbalanced electron-wave functions between the two sulfur atom-layers. Moreover, the two-dimensional atomic-resolved polar (specifically as Janus TMD) monolayers with an intrinsic atomic inversion asymmetry enrich the Rashba SOC family and facilitate the development of spin-field-effect transistors. The exceptional spin-precession in Janus TMD monolayers can be electronically controlled in a precise and predictable manner applying for spin-field effect transistors.

Theoretical and experimental studies demonstrate that MoS_2_ and MoSe_2_ monolayers can be modified to the sandwiched S–Mo–Se Janus structure by atomic exchange, resulting in a key transition in lattice dynamics and electronic properties. It is thus predictable and feasible to obtain the inversion asymmetric 2D topological insulators used for artificial spintronic manipulations by exfoliating from the covalently bonded multi-layers of 2D sulfur compounds or posterior 2D materials [19,20]. To date, there has been no systematic study in previous reports on the essential cause and mechanism for SOC-introduced and spin-polarized spin splitting in the electronic structures of monolayered Janus TMD structures. Recent experimental syntheses of 2D vanadium(V)-dichalcogenide compounds as for VS_2_ and VSe_2_ monolayers, which can convert from semi-semiconductor to semi-metal over a wide doping density range and where electrons and holes share a spin channel, suggest potential new schemes for developing prospective functional materials applied in nanoelectronics. Due to the complex and variable electronic structures of 2D monolayered TMD compounds, the single layers of V-dichalcogendies (monolayers) deserve special studies as a 2D material with both spin topology and controllable magnetism. Therefore, in order to further extend the preferable applications of 2D VX_2_ (X = S, Se, Te) monolayers in nanoelectronics and spintronics, it is crucial and of high interest to study the spintronic structure and magnetism of Janus compound VXY (X,Y = S, Se, Te) monolayers. In the present study, we employ the first-principles pseudopotential plane-wave method to theoretically investigate the structural and spintronic properties of Janus VXY monolayers, whilst evaluating and analyzing the dynamic stability and bonding characteristics by phonon structure and electron density difference, respectively. We focus on the SOC and polarized spin splitting in attribute to atomic orbital components of electronic states near energy band-edge, estimate the spin magnetic moment and elucidate the spin coupling orders.

## 2. Theoretical Methodology

Conforming to pseudopotential plane-wave first-principles method based on spin density functional theory, the atomic structure, phonon dispersion, electronic structure and magnetic characteristics of Janus vanadium-dichalcogenide monolayers are calculated as implemented in CASTEP code of Materials Studio 2020 software package (Accelrys Inc., *Materials Stutio* version 2020.08, San Diego, CA, USA). Gradient-corrected exchange-correlation functional of PBESOL form is adopted to perform the spin-polarized calculations, in which the spin-up and -down states described by different wave-functions are calculated according to relativistic Dirac equations [21]. Non-collinear spin-polarization and SOC are incorporated into the energy, geometry, charge population, electron density difference and electrostatic potential calculations. In order to elucidate SOC-introduced spin splitting, the non-polarized band structures with and without SOC are calculated for comparisons. Collinear spin-polarization is specified to calculate the spin-resolved electron density, band structure, density of energy states and net spin for investigating the space asymmetry of spin distributions and the resulting magnetism.

The interactions between atomic cores and electrons are modeled by norm-conserving pseudopotential, which incorporates treating relativistic effects in Koelling–Harmon scheme [22]. Electronic-wave functions are expanded by plane-wave basis set with an energy cutoff in 1220 eV. Finite basis-set correction is always applied to energy and stress calculations by estimating energy derivatives using numerical differentiation on 3 points [23]. Self-consistent field (SCF) iterations are carried out with a convergence tolerance of 5 × 10^−7^ eV/atom in a FFT grid of 36 × 36 × 320 to ensure the accuracy of electron density calculations, in which Pulay scheme of density mixing in charge and spin magnitudes of 0.5 and 2.0 respectively is utilized to realize electron relaxations [24,25]. The ***k*** point sampling of integration in Brillouin zone (BZ) is carried out on Monkhorst–Pack 5 × 5 × 1 grid [26]. 

Atomic-structured models are constructed in 3-dimension by setting a sufficiently thick vacuum layer (30 Å) between the periodic-imaged molecular layers so that the actual representation is a two-dimensional monolayer. Geometry optimizations for relaxing the initial atomic structure are performed by minimizing energy functional with LBFGS algorithm in delocalized internal coordinates, to reach an energy convergence of 5.0 × 10^−6^ eV/atom with the atomic force and stress being less than 0.02 eV/Å and 0.001 Å, respectively [27]. To confirm the structural stability derived from cohesive energy calculations, we also calculate the elastic coefficient of in-plane (parallel to layer-plane) biaxial tensile strain as formularized by Δ*E*_unit_ = *k*(Δ*a*/*a*)^2^/2, where Δ*E*_unit_ denotes total energy increment per unit-cell under deformation from optimized geometry, *k* symbolizes elastic coefficient, *a* is 2D unit-cell lattice constant of geometry-optimized monolayer, Δ*a*/*a* represents biaxial strain in V-dichalcogenide monolayers. Employing the identical DFT scheme as geometry optimizations, the total energies of monolayers under various strains (maximum strain amplitude is specified as 0.005) are calculated to estimate elastic coefficients by the second derivative of the total energy to geometry deformation. Finite-displacement technique with a force constant convergence criterion of 1.0 × 10^−5^ eV/Å^−2^ and OTFG (on the fly generation)- Ultrasoft pseudopotential are used to calculate phonon structures (frequency dispersion) of geometry-optimized monolayers under collinear spin polarization. Furthermore, LO-TO splitting is evaluated by a non-spin-polarized scheme to speculate if it is significant to be incorporated into phonon calculations. 

## 3. Results and Discussion

### 3.1. Atomic Structure and Stability

**Figure 1:**
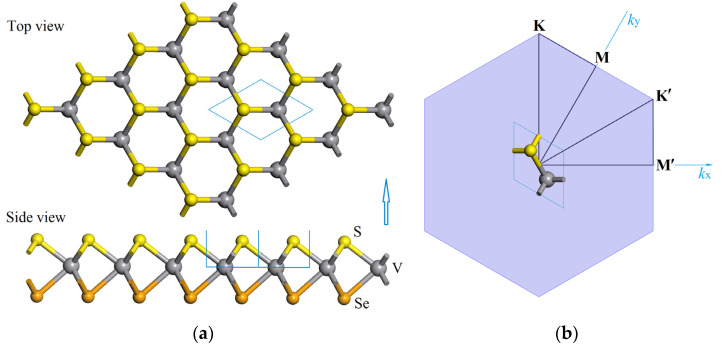
(**a**) Top view (top) and side view (bottom) of VSSe monolayer in the relaxed structure after geometry optimization, with the yellow, gray and orange balls symbolizing S, V and Se atoms, respectively; (**b**) specific high symmetry points (M, K, M′, K′) along the dispersion paths of phononic frequency or electronic energy in the first Brillouin zone.

A monolayered VXY (X,Y = S, Se, Te) compound is a typical Janus 2D structure while retaining the in-plane structural symmetry of a binary VX_2_ monolayer. In the V-sandwiched Janus structure of VXY monolayer, the V and X/Y atoms form a hexagonal 2D lattice in which three X and three Y atoms bond chemically with V atoms on both sides of V atomic layer into a triprism structure, as Figure 1 shows for the side and top viewing of the relaxed atomic structure of VSSe monolayer. The periodic atomic structure of VXY monolayers with a space symmetry of P3m1 group belongs to a trigonal crystal system, while it lacks mirror symmetry compared to VX_2_ monolayers (P6m2 space group). The crystal lattice constant, V-chalcogen bonding length, layer thickness (distance between two chalcogen atoms), conhesive energy, and Mulliken atomic charges obtained from first-principles calculations of VXY and VX_2_ (for comparison) monolayers are listed in Table 1. The lattice constant, bonding length and layer thickness of vanadium-dichalcogenide monolayers can be distinguished merely by complying with atomic number or atomic radius in order. Furthermore, the cohesive energy also decreases uniformly with the increase in atomic number/radius for both VX_2_ and Janus VXY monolayers, and approaches the highest and lowest values of 15.59 and 11.77 eV/unit-cell (averaged 5.16 and 3.59 eV/atom) for VS_2_ and VTe_2_ monolayers, respectively, which are completely higher than that of III-chalcogenide covalent double-layers, such as GaSe, InSe and Janus GaInSe_2_ [28,29,30]. It is an energetic manifestation that the high structural stabilities of vanadium-dichalcogenide monolayers can be acquired by V-dichalcogen bonding strongly into a perfect crystalline structure. The cohesive energies of VXY monolayers are similar to the averaged values of their two counterparts with identical chalcogens, which could be described by *E*_coh_(VXY) ≈ *E*_coh_(VX_2_)/2 + *E*_coh_(VY_2_)/2. In addition, the elastic coefficients of VSTe and VTe_2_ monolayers under biaxial strain are evidently higher compared with the other monolayered V-dichalcogenides, as listed in Table 1, implying their higher mechanical stability of resisting the hydrostatic deformations that need applying higher stresses. 

**Figure 2:**
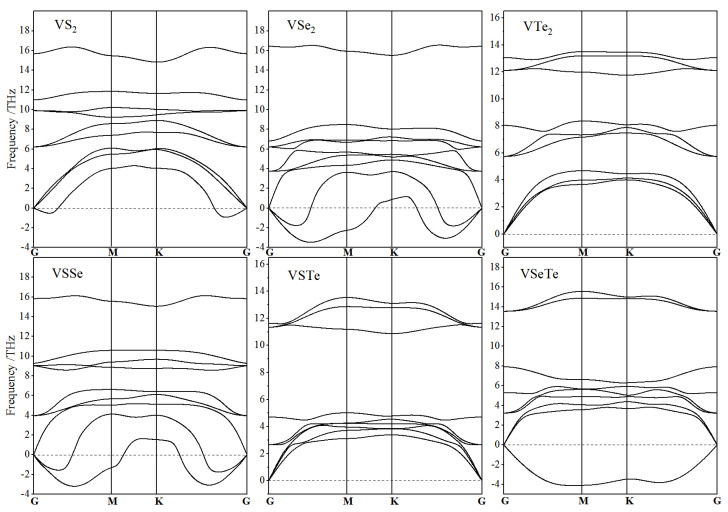
Phonon structures (frequency dispersion spectra) of vanadium-dichalcogenide monolayers.

By finite-displacement technique, the phonon frequencies can be calculated without limitations on physical properties of crystal materials, which is comprehensively available for metal and spin-polarization systems, whereas for both the linear response and finite-displacement techniques, the LO-TO splitting is only restricted on non-spin-polarized calculations of phonon structures. Accordingly, LO-TO splitting phonon structures are calculated for vanadium-dichalcogenide monolayers to provide a probative verification that it could be a good approximation to ignore LO-TO splitting. The degenerate frequencies of LO-TO wave couples without any splitting at Brillouin zone center for all the vanadium-dichalcogenide monolayers under non-polarization verify that it is viable and preferable of incorporating spin polarization to calculate chemical-bonding forces for atomic displacements without considering LO-TO splitting. In order to precisely evaluate the structural stability under atomic oscillations, the phonon structures of vanadium-dichalcogenide monolayers are calculated with the inclusion of collinear spin polarization, as shown in Figure 2.

Since the unit-cell of VXY monolayer consists of one vanadium atom and two different sulphate atoms, its phonon dispersion spectrum represents nine branches, including three acoustic branches and six optical branches, as shown in Figure 2. From high-to-low frequencies, acoustic phonons are classified into longitudinal acoustic waves (LA), in-plane transverse acoustic waves (TA), and out-of-plane acoustic transverse waves (ZA). The VXY monolayer possesses two sets of optical phonons, each containing a non-degenerate off-plane transverse optical wave (ZO) and a pair of G-point degenerate longitudinal optical wave (LO) and in-plane transverse optical wave (TO), respectively. In the six vanadium-dichalcogenide monolayers, only VTe_2_ and VSTe monolayers show sheerly positive intrinsic-frequencies of phonon modes, without any virtual frequency in low-frequency acoustic waves (usually arising in 2D structure), implying that they are dynamically stable 2D structure, which is consistent with their higher elastic coefficients under in-layer strains. It appears that V–Te bonding can present a higher stability in audio wave modes and even could cancel out all the unstable ZA phonons from V–S bonds near G-point, as comparatively manifested by the phonon structures of the VS_2_, VTe_2_, and VSTe monolayers. In contrast, almost half of audio phonon modes are imaginary with a negative frequency in both VSe_2_ and VSSe monolayers, implying the high instability of V–Se bonds in audio lattice dynamics. 

### 3.2. Internal Gradient of Electrostatic Potential

**Figure 3:**
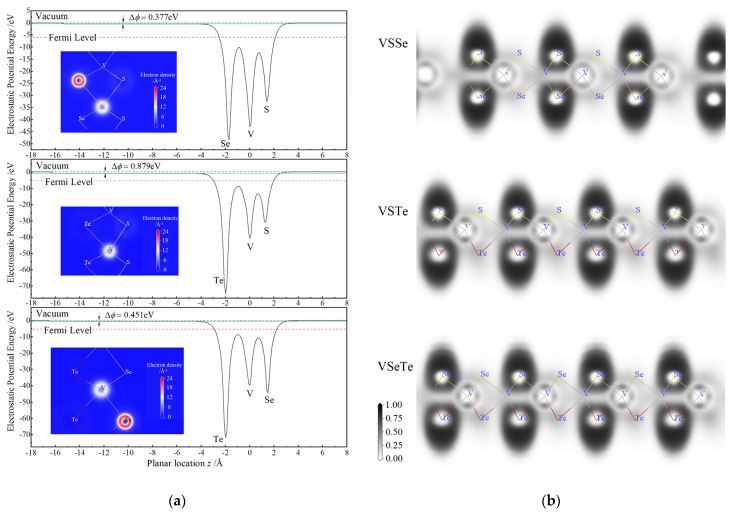
Internal electric field and bonding characteristics of Janus V-dichalcogenide monolayers: (**a**) the averaged electrostatic-potential energies on the layer-plane as a function of *z*-axis coordinate that is perpendicular to layer-plane and marking its position away from V atomic center; (**b**) electron localization function contoured with colors on the plane vertical to layer and crossing both V and chalcogen atomic centers.

Mulliken charges populated on Te atoms by positive 0.2~0.3e due to the unique less electronegativity of Te element than V element imply the specific charge transfers entirely from V and Te to S or Se in VTe_2_, VSTe and VSeTe monolayers, in contrast to the negative atomic charges of −0.8~−1.2e on S and Se atoms, respectively, in the other vanadium-dichalcogenide monolayers. As a result, compared with VSSe monolayer, an evidently higher gradient of electrostatic potentials in VSTe and VSeTe monolayers is engendered by a large discrimination in the atomic population charges of 1.47e and 1.38e, respectively, as illustrated in Figure 3a, in which the electrostatic-potential energy differences of ∆*ϕ* = 0.377, 0.451 and 0.879 eV between two different chalcogen surfaces are caused, respectively, for the VSSe, VSeTe and VSTe monolayers. In confirmation, the electron densities contoured by blue-white-red maps on the layer-plane through the bonding atoms are also shown in the inserted panels of Figure 3a. Therefore, it is suggested that the mirror symmetry as in VX_2_ monolayers are broken distinctively in the VSTe and VSeTe monolayers, which is attributed to the neutral area (zero electrostatic potential) plane and electron clouds shifting towards one chalcogen surface of S or Se with a much higher electronegativity than Te atoms. In contrast, the remarkable charge transfer from V atomic plane to both S and Se surfaces characterizes similar ionic-bonding in VSSe monolayer as VS_2_ or VSe_2_ monolayer, leading to a minimal discrepancy between the population charges on S and Se atoms as shown in Table 1, despite their rather distinctions in electronegativity. Accordingly for Janus vanadium-dichalcogenide monolayers, the electronegativity of V atoms and the positive population charges on chalcogen atoms could be referenced as a judgement of the substantial intrinsic electric field (internal electrostatic-potential gradient) perpendicular to layer-plane.

Bonding characteristics of V with chalcogenide atoms are revealed by the electron localization function (ELF) deriving from SCF electron density in space, which are contoured by the color maps on the plane crossing atomic centers and perpendicular to monolayer sheet, as shown in Figure 3b. For V–S and V–Se bonds of all three Janus V-dichalcogenide monolayers, the highest ELF value arises near V and S/Se atomic cores, as a manifestation of ionic bonding character by a significant electron transfer from V to S or Se atoms. For V–Te bonding, in contrast, the high ELF region around Te center extends approaching V locate and stretches over the middle between V and Te centers, thereby showing a partial covalent feature of pairing electronic-states from both bonding atoms, which also accounts for a significant electrostatic-potential gradient between the Te and S/Se atomic layers. In consistence with Mulliken charge populations and electrostatic potentials, the ELF distributions elucidate the chemical bonding attributes of the internal electric fields caused by atomic inversion asymmetry in Janus V-dichalcogenide monolayers.

### 3.3. Band Structure 

**Figure 4:**
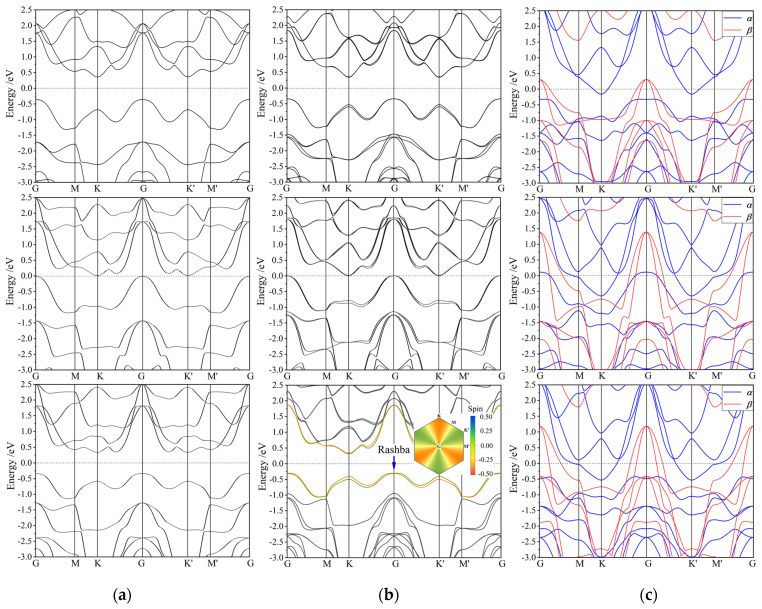
Band structures of VSSe (top panels), VSTe (middle-row panels), VSeTe (bottom panels) monolayers: (**a**) without SOC, (**b**) with SOC well determined by non-polarization (the inserted Brillouin zone is color contoured by spin compositions of the highest valence-band electrons), and (**c**) spin resolved by collinear spin polarization, in which Fermi level is referenced as energy zero indicated by the horizontal dash line.

In graphene, Rashba spin splitting relies on the SOC strength as well as on the external electric field perpendicular to the graphene plane, while there is still no experimental observation of Rashba splitting in pristine graphene or silicene due to the weak SOC for C and Si [9,10]. It has been demonstrated by ab initio calculations that Rashba spin splitting arises in all the Janus Mo and W dichalcogenide monolayers, and can be enhanced by stronger SOC and the internal potential gradient. In contrast, for Janus vanadium-dichalcogenide monolayers, the spin-orbit coupling of vanadium element is much weaker than Mo and W, and S atom has almost no SOC. Therefore, Rashba spin-splitting occurs merely in the VSeTe monolayer with a Rashba parameter *R*_G_ of 0.658 eV·Å due to the strong SOC from both Se and Te atoms, as illustrated by the lateral splitting arising at G point only in SOC band structure of VSeTe monolayer (Figure 4b bottom panel) by contrast to non-SOC band structures (Figure 4a). It can be inferred by the highest built-in electric fields existing in non-Rashba VSTe monolayer that strong SOC dominates the engendering of Rashba splitting in Janus TMD monolayers, requiring both two sulfur elements with strong SOC to produce a significant Rashba splitting, as is true for Janus Mo or W dichalcogenide compounds. Meanwhile, SOC also causes remarkable band-edge splittings at K point for all the three Janus V-dichalcogenide monolayers, which is explicitly larger for a higher atomic number of dichalcognen elements, as indicated by *λ*v and *λ*c for the upmost valence band and downmost conduction band, respectively, in Figure 4b and the values that are listed in Table 2. For VSSe and VSeTe monolayers with a preferable valley shape of electronic energy dispersion rather than the flat band of VSTe monolayer at K point, the upmost valence band with a much larger *λ*v is more promising for valleytronic applications rather than the downmost conduction band with a minimal *λ*c [13]. Under the quantum representation of SOC or non-collinear spin polarization, the spin-up and spin-down states cannot be strictly distinguished since the quantum angular momentum is not a good quantum number, as shown for the Rashba VSeTe monolayer in the bottom panel of Figure 4b in which the mixed sates of two different spin components are color contoured in the electronic bands near Fermi level, which are also mapped on Brillouin zone for the highest valence band.

To explore the spin space asymmetry and magnetic order due to spin polarization, the spin-resolved band structure was calculated with collinear spin polarization, as shown by the results in Figure 4c. Spin polarization leads to remarkable spin splittings near Fermi level for the monolayered Janus vanadium-dichalcogenides: the spin-up (α) electronic states of conduction band minimum (CBM) at K point are lower in energy than the spin-down (*β*) electronic states of G-point valence band maximum (VBM), representing a semi-metallic character. Hence, under the assistants of phonons, a large number of electrons near G-point VBM transit to CBM at M point, leading to the Fermi level residing at the middle between G-point VBM and K-point CBM, which implies a metallic conductance in layer-plane. In the optoelectronic transitions, it requires the energy, momentum and spin-state conservations (Fermi-golden rule), so the photon-absorption edge is determined by the spin-conserved vertical band-gap (energy for absorbing or emitting photons) in band structure, which could be annotated by optoelectronic band-gap. For all three monolayered Janus vanadium-dichalcogenides, the optoelectronic band-gaps obtained from spin-resolved band structures are listed in Table 2.

Due to the collinear spin polarization, both the lowest conduction band (CB1) and the second-upmost valence band (VB2) split distinctly into the lower-level spin-up (*α*) state and the higher-level spin-down (*β*) state, as shown in Figure 4c. Accordingly, both the *α*-CB1 and *β*-VB2 bands disperse across Fermi level, especially for VSTe and VSeTe monolayers. This significant spin splitting near Fermi level leads to an evidently higher number of *α*-state electrons than *β*-state electrons in valence band, which accounts for the acquisition of a considerable net spin (intrinsic magnetic moment) in Janus V-chalcogenide monolayers, as shown in Table 2. The spin splitting near Fermi level and the resulting net spin increase significantly with the atomic number of sulfur elements, indicating the considerable contributions from sulfur atomic *p*-orbitals to Fermi-level spin polarization in the monolayered Janus vanadium-dichalcogenides. Both VSTe and VSeTe monolayers can present a considerable net spin of ~1.63 *ћ*/2 (μB), which is evidently larger than 1.16 *ћ*/2 of VSSe monolayer, as shown in Table 2. This result can be proportionally attributed to the spin-polarized energy splitting near Fermi level, with the dominant and minor components derived respectively from V-3*d* and Te-5*p* orbitals.

### 3.4. Magnetic Property

**Figure 5:**
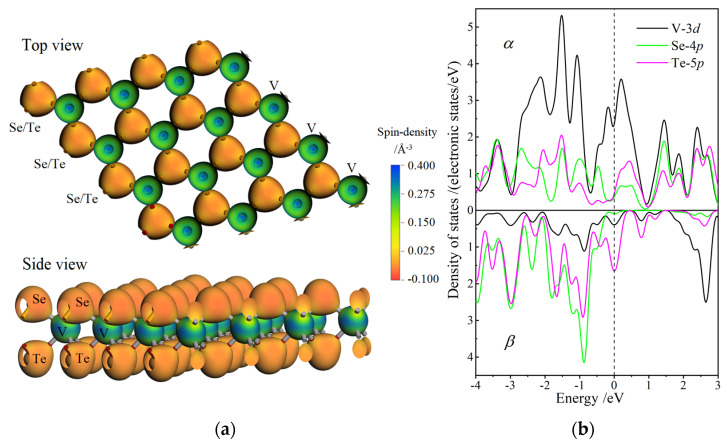
(**a**) Spin density contoured by color maps on electron density isosurfaces of 0.4 electrons/Å^3^ in VSeTe monolayer; (**b**) spin-dependent partial atomic-projected DOS of VSeTe monolayer for V-3*d*, Se-4*p* and Te-5*p* orbitals where the vacuum free electron energy level is referenced as energy zero.

For transition-metal dichalcogenide monolayers, the inversion symmetry of atomic geometry diminishes in the direction normal to layers, while it still persists in layer-plane. Nevertheless, it is very possible for spin polarization to break the in-plane inversion symmetry of spin-resolved electronic states and result in energetic spin splitting at Fermi level. Therefore, for Janus V-dichalcogenide monolayers, the spin electron density in real space and the spin-dependent orbital-resolved (partial) atomic-projected density of states (DOS) are calculated by collinear spin polarization to investigate the spin asymmetry magnetic order in layer-plane and the atom-orbital contributions to spin magnetic moment. As a representative manifestation of the ferrimagnetic spin coupling between V and chalcogen cations, the VSeTe monolayer presents the largest net spin of 2.25 *ћ*/2, which can be traced to the major sin-polarized contribution from V-3*d* orbital component, as shown by the band structures and net spin values in the previous section. Moreover, due to the similar chemical circumstance near V cations, all the vanadium-dichalcogenide monolayers represent a similar magnetic characteristics, energetically originating from the spin splitting near Fermi level. Emblematically for Janus V-dichalcogenide monolayers, the spin asymmetric distribution and spin-splitting origination of VSeTe monolayer are illustrated by the spin density distribution (on the isosurface of total electron density) and the spin-dependent partial atomic-projected DOS, as respectively shown in Figure 5a,b.

On the isosurface of the total electron density, the major *α* and *β* components of spin-polarized electrons are appreciably distinguished by positive and negative spin densities around V and chalcogenide locates, respectively, implying that the spin symmetry between *α* and *β* electrons has been destroyed by the spin polarization in layer-plane. The majority of electrons are spin-polarized into *α*-states and *β*-states respectively residing at the local spaces of V and Se/Te atoms. In particular, the higher absolute value of *α*-dominated spin density around V atoms than that of *β*-dominated electrons around Se/Te atoms indicates the more *α* electrons than *β* electrons in the entire monolayer, as manifested by a significant net spin listed in Table 2. In layer-plane, it is also implied that ferromagnetic spin polarization between V cations leads to a considerable spin magnetic moment, while dichalcogenide and vanadium cations are coupled in a spin ferrimagnetic order, as shown in Figure 5a. The spin magnetic moment originates dominantly from spin-polarized components of V-3*d* and Te-5*p* orbitals with an energetic-preferential spin splitting near Fermi level, as shown in Figure 5b. Accordingly, the density of spin-polarized electronic states is mightily abated by forming the ionic V bonding with a higher electronegativity of chalcogen atoms. Therefore, VSTe and VSeTe monolayers presents a distinctly larger net spin than that of VSSe monolayer, in consistence with the positive Mulliken charges acquired uniquely by Te cation compared to the negative-charged S and Se cations (as shown in Table 1).

## 4. Conclusions

To this end, we accomplished a systematic study on the spin-polarized electronic structures of Janus vanadium-dichalcogenide VXY (X, Y = S, Se, Te) monolayers, by the first-principles pseudopotential plane-wave method. Even though the cohesive energies obtained from geometrically optimized structures of VTe_2_ and VSTe monolayers are relatively lower than the other V-dichalcogenides, the phonon dispersion curves demonstrate that they are stable in atomic-vibration dynamics, being consistent with their significantly higher elastic coefficients under in-layer biaxial strain. Rashba splitting originates dominantly from the strong SOC of both V and chalgen atoms, as only represented by VSeTe monolayer with a minimal potential gradient perpendicular to layer-plane. The spin order in layer-plane and the SOC-induced splitting at K point suggest the potential applications of Janus V-dichalcogenide monolayers in spintronics and valleytronics. The larger spin magnetic moment of VSTe and VSeTe monolayers than that of VSSe monolayer is attributed to the Fermi-level spin-splitting components of both V-3*d* and Te-5*p* orbitals.

## Figures and Tables

**Table 1:** Space symmetries, lattice constants *a*, V–chalcogen bonding lengths *d*_VX_ and *d*_VY_, distance between two chalcogen cations on the two surface sides of monolayer (layer thickness, *d*_XY_), cohesive energy *E*_coh_, Mulliken atomic charge *q*_M_, and biaxial elastic coefficient *k* of VX_2_ and VXY monolayers.

Space Group	V_XY_ Monolayer	*a*/Å	*d*_VX_ and *d*_VY_/Å	*d*_XY_/Å	*E*_coh_/(eV/Unitcell)	*q*_M_/e			*k*/(eV/Unitcell)
						V	X	Y	
P6*m*2	VS_2_	3.136	2.338	2.959	15.5893	1.86	−0.91	−0.91	167.6
	VSe_2_	3.295	2.480	3.183	14.1387	1.82	−0.89	−0.89	186.5
	VTe_2_	3.551	2.691	3.488	11.7610	−0.30	0.19	0.19	277.8
P3*m*1	VSSe	3.223	2.327, 2.490	3.052	14.9025	1.93	−1.06	−0.82	203.6
	VSTe	3.356	2.319, 2.711	3.170	13.6388	1.00	−1.20	0.26	254.8
	VSeTe	3.421	2.476, 2.703	3.339	12.9642	0.81	−1.06	0.31	173.9

**Table 2:** SOC-induced splitting *λ*v and *λ*c at K point for valence and conduction band-edges, respectively, Rashba spin splitting *R*_G_ of the highest valence band at G point, optoelectronic band-gap *E*_og_, and net spin *s*_n_ of Janus V-dichalcogenide monolayers.

VXY Monolayer	*λ*v/meV	*λ*c/meV	*R*_G_/eV·Å	*E*_og_/eV	*S*_n_/(*ћ*/2)
VSSe	68	11	0	0.93	1.16
VSTe	85	19	0	0.09	1.63
VSeTe	92	22	0.658	0.15	1.64

## Data Availability

Theoretical methods and results are available from the authors.
